# Characterization and antibacterial efficacy of *Streptomyces* sp. NELs-40 against *Staphylococcus aureus*

**DOI:** 10.3389/fmicb.2026.1840366

**Published:** 2026-05-29

**Authors:** Mamy Jayne Nelly Rajaofera, Xuemiao Li, Wenqing Wang, Yutian Qin, Yuanyuan Li, Chun Jiang, Jingsi Jiang, Dai Kuang

**Affiliations:** 1 NHC Key Laboratory of Tropical Disease Control, School of Life Sciences and Medical Technology, Hainan Medical University, Haikou, Hainan, China; 2 School of Hainan Provincial Drug Safety Evaluation Research Center, Hainan Medical University, Haikou, Hainan, China

**Keywords:** antibiotic resistance, biofilm inhibition, biosynthetic gene clusters, MRSA, secondary metabolites, *Streptomyces parvus*

## Abstract

**Background:**

Methicillin-resistant *Staphylococcus aureus* (MRSA) presents a critical therapeutic challenge, and *Streptomyces* species are promising sources of novel antimicrobials.

**Objective:**

This study aimed to characterize *Streptomyces* sp. strain NELs-40 genomically, optimize its antimicrobial production, evaluate its efficacy against MRSA ATCC 43300, and identify its bioactive metabolites.

**Methods:**

Genomic DNA was sequenced (Illumina HiSeq X10) and assembled (SOAPdenovo2). Taxonomic classification was determined through 16S rRNA, ANI, AAI (CompareM), and dDDH (TYGS). Synteny analysis was performed using Clinker. Biosynthetic gene clusters (BGCs) were predicted with antiSMASH v4.2.0. Antimicrobial production was optimized via Plackett-Burman design. Antibacterial activity was assessed through well diffusion, MIC determination, and anti-biofilm assays. Bioactive metabolites were analyzed by GC–MS and tested for thermal, pH, and enzymatic stability.

**Results:**

Genomic analysis revealed a complete genome of 7,973,786 bp with 71.76% GC content, harboring 6,872 protein-coding genes and 49 biosynthetic gene clusters (BGCs). Of these, 30 clusters were associated with antimicrobial compound biosynthesis, representing diverse classes including polyketides and nonribosomal peptides. Taxonomic classification was confirmed by comparison with two reference genomes. Against the *S. parvus* reference genome, NELs-40 exhibited ANI of 99.6%, AAI of 98.31%, and dDDH of 88.70%. Against *S. parvus* A612, the values were ANI of 98.50%, AAI of 98.77%, and dDDH of 90.70%. Statistical optimization identified pH as the primary factor affecting antimicrobial production (*p* < 0.05). The ethyl acetate extract demonstrated potent activity against MRSA ATCC 43300, producing inhibition zones of 37 mm, an MIC of 125 μg/mL, and 32.25% biofilm inhibition. GC–MS analysis identified 23 compounds, predominantly 9-octadecenoic acid methyl ester, pyrrolo[1,2-a]pyrazine-1,4-dione derivatives, caryophyllenyl alcohol, and sesquiterpenes. The extract maintained optimal activity at pH 6.0–7.0 and temperatures up to 40 °C, with significant activity loss above 60 °C. The extract also demonstrated resistance to proteolytic enzymes, indicating non-proteinaceous characteristics.

**Conclusion:**

*Streptomyces* sp. strain NELs-40 is a promising source of novel antimicrobial agents against drug-resistant pathogens, with extensive biosynthetic capacity, diverse chemistry, and potent activity against MRSA.

## 1 Introduction

*Staphylococcus aureus* has emerged as one of the most challenging pathogens in clinical practice, capable of causing infections ranging from superficial skin lesions to severe diseases such as sepsis, pneumonia, and endocarditis (Lowy, 1998; Turner et al., 2019). Its rapid resistance development has transformed this pathogen into a critically difficult to treat in many clinical scenarios (Vestergaard et al., 2019; Lin et al., 2024). The emergence of methicillin-resistant *S. aureus* (MRSA) has significantly compromised the therapeutic options, leaving clinicians with severely limited options and escalating mortality rates (Álvarez et al., 2019; Cong et al., 2020; Jubeh et al., 2020). Traditional antibiotics are becoming ineffective at an accelerating rate, necessitating the urgent discovery of novel antimicrobial agents (Shelke et al., 2023; The Lancet, 2024).

*Streptomyces* species are prolific producers of secondary metabolites, many of which exhibit potent antimicrobial activities (Hu et al., 2021; Del Carratore et al., 2022; Parra et al., 2023). These gram-positive bacteria have revolutionized medicine through cornerstone antibiotics like streptomycin, tetracycline, and erythromycin, while demonstrating broad-spectrum antibacterial, antifungal, and anticancer activities (Manhas et al., 2022; Parra et al., 2023). Their metabolic versatility positions them as exceptional resource for next-generation antibiotic discovery.

This study characterizes *Streptomyces* sp. strain NELs-40, isolated from mangrove rhizosphere soil, which demonstrates potent inhibitory activity against *S. aureus*, including drug-resistant strain. Our objectives are to isolate and characterize the active metabolites, determine their antimicrobial spectrum, and evaluate their therapeutic potential against clinically relevant *S. aureus* strains, particularly MRSA.

## 2 Materials and methods

### 2.1 Microorganisms and growth conditions

The antimicrobial strain NELs-40, isolated from mangrove rhizosphere soil in Hainan province, China, and the MRSA strain from the laboratory culture collection were used in this study. Both strains were preserved at −80 °C in glycerol stocks (40% glycerol; Xilong Scientific Co., Ltd., China). NELs-40 was cultured in tryptic soy broth (TSB; Hopebio, Qingdao, China) at 28 °C for 5 days, while MRSA was grown Luria–Bertani (LB) medium prepared according to standard formulations at 37 °C for 24 h prior to experimental use (Boxun Medical Biological Instrument Corp., Shanghai, China).

### 2.2 Bacterial identification and genome characterization

NELs-40 was previously identified as *Streptomyces* sp. through 16S rRNA gene sequencing analysis. The genome sequencing and annotation were subsequently performed to further characterize this strain. NELs-40 was cultured in the TSB medium (Hopebio, Qingdao, China) at 180 rpm for 72 h at 28 °C (Boxun Medical Biological Instrument Corp., Shanghai, China). After centrifugated (3,000 rpm for 5 min, Sigma Laborzentrifugen GmbH, Germany), genomic DNA was isolated using the Omega Genomic DNA Purification Kit (Omega Bio-tek, United States). The whole genome sequencing and assembly were carried out by the Majorbio Bio-pharm Technology Co., Ltd. (Shanghai, China). The Illumina Hiseq × 10 Platform (Illumina, United States) was used for sequencing, and the raw data for assembling was more than 100× of genome coverage depth. Sequence assembly was performed using the SOAPdenovo2 (Luo et al., 2012). The protein-coding genes and gene functions were annotated as previously described (Jing et al., 2020; Zhou et al., 2022). The predicted genes were annotated by different databases of NR (Sayers et al., 2022), Swiss-Prot (Bateman et al., 2022), Pfam (Mistry et al., 2020), GO (Carbon et al., 2020), COG (Galperin et al., 2020) and KEGG (Kanehisa et al., 2020). Biosynthetic gene clusters (BGCs) of secondary metabolites were predicted by the antiSMASH v4.2.0 software (Majer et al., 2021).

For taxonomic classification, the average nucleotide (ANI) was calculated for the further identification of strain classification status (Richter and Rosselló-Móra, 2009) to compare NELs-40 with reference *Streptomyces* genomes. Additionally, Average Amino Acid Identity (AAI) was calculated using CompareM with the aai_wf command. Genomic nucleotide sequences in FASTA format were used as input, with genes predicted *de novo* using Prodigal (Hyatt et al., 2012). Reciprocal best hits were identified using DIAMOND (Buchfink et al., 2015) with default parameters. AAI values exceeding the 95–96% species delineation threshold were considered indicative of species-level classification (Richter and Rosselló-Móra, 2009). The Type Strain Genome Server (TYGS) was used for whole-genome-based taxonomic analysis, including digital DNA–DNA hybridization (dDDH) calculations (Meier-Kolthoff and Göker, 2019). Synteny analysis was performed using Clinker v1.0.0 (Gilchrist and Chooi, 2021) to compare the genome organization of NELs-40 with closely related reference genomes. Clinker generates interactive synteny plots by identifying and visualizing collinear regions and homologous gene clusters between multiple genomes.

### 2.3 Antimicrobial activity assay and optimization of culture conditions

Antibacterial activity of NELs-40 was determined using the well diffusion method. Test pathogen was cultured in their respective media (Supplementary Table S1) at 37 °C for 24 h using an incubator (Boxun Medical Biological Instrument Corp., Shanghai, China). A 100 μL aliquot of fresh culture was mixed with 10 mL of melted agar medium (44 °C), inoculated onto plate surfaces, and incubated at 37 °C under same condition. Inhibition zones were observed after 48 h.

Culture conditions for antimicrobial production were optimized using Plackett-Burman design with Design-Expert software (Stat-Ease Inc., Minneapolis, MN, United States). Five independent variables (Supplementary Table S2) including, temperature, pH, agitation speed, inoculation time, and inoculum size were tested at high (+1) and low (−1) levels across 12 trials conducted in triplicate (Supplementary Table S3). Antibacterial activity against MRSA ATCC 43300 was measured by inhibition zone diameter as the response variable. Data were analyzed at 5% significance level (*p* < 0.05) using the regression equation: *Y* = β₀ + β₁*X*₁ + β₂*X*₂ + ⋯ + βₙ*X*ₙ, where *Y* is the predicted response, β₀ is the intercept, β₁, β₂ to βₙ are variable coefficients, and *X*₁, *X*₂ to *X*ₙ are coded values of independent variables. Model adequacy was determined through ANOVA, and quality was assessed using coefficient of determination (*R*^2^) and adjusted *R*^2^. Optimum variable levels were determined using the software’s response optimizer tool.

### 2.4 Production and extraction of bioactive compounds

NELs-40 was cultured in broth at 28 °C for 5 days using an incubator (Boxun Medical Biological Instrument Corp., Shanghai, China), and cell-free supernatant was obtained by centrifugation (10,000 × g for 10 min, Beckman Coulter, United States). The supernatant was extracted with ethyl acetate (1:1, v/v; Xilong Scientific Co., Ltd., China), and the organic layer was collected and evaporated to dryness using a rotary evaporator (Heidolph Instruments GmbH & Co. KG, Germany). The extract was further freeze-dried using a lyophilizer (Scientz-10 N, Scientz Biotechnology Co., Ltd., Ningbo, China). A control extract was similarly prepared from non-inoculated sterile broth to confirm that any observed antibacterial activity originated from the bacterial metabolites. The dried extract was dissolved in DMSO (2 mg/mL; Macklin, China) and filter-sterilized through a 0.22 μm membrane (Jinteng Experimental Equipment Co., Ltd., China) before being tested for antibacterial activity using the well diffusion method. Vancomycin (WeiShi Reagent Shanghai, China) is used as positive control.

### 2.5 Thermal, pH, and enzymatic stability testing

The heat stability of the crude extract was determined using different range of temperature (40 °C–121 °C) for 30 min. For pH stability, the compound was incubated over pH range of 2.0–12.0 for 30 min at 37 °C. The sensitivity of the extract to enzymes was also tested against proteinase K, trypsin, pepsin, and lypase (Omega Bio-tek, United States). Antibacterial activity was determined by well diffusion method (Rajaofera et al., 2018).

### 2.6 Assessment of antibiofilm activity

Anti-biofilm activity was evaluated using microtiter plate assay. The crude extract was prepared according the method described above. Test strain (10^8^ CFU) was inoculated into plates containing TSB (Hopebio, Qingdao, China) and the extract, then incubated at 37 °C for 24 h using an incubator (Boxun Medical Biological Instrument Corp., Shanghai, China). Biofilms were fixed with methanol (Xilong Scientific Co., Ltd., China), stained with 0.5% crystal violet (Solarbio, China), solubilized with 30% glacial acetic acid (Xilong Scientific Co., Ltd., China), and measured at 595 nm (Tecan 350VA, Tecan Group Ltd., Männedorf, Switzerland). Biofilm inhibition was calculated as: % inhibition = (A₅₉₅ control - A₅₉₅ treated/A₅₉₅ control) × 100. Results represent mean ± SEM from four experiments.

### 2.7 Gas chromatography (GC–MS) analysis

The active extract was analyzed using GC–MS system (7890B GC coupled with a 7000B Triple Quadrupole MS, Agilent Technologies, Santa Clara, CA, United States). Data acquisition was performed using MassHunter GC/MS Acquisition software (Agilent Technologies, United States). The components were preliminarily identified by comparing their retention times and mass spectra with those in the NIST98 library (National Institute of Standards and Technology, Gaithersburg, MD, United States), as well as with published literature data (Zhou et al., 2022).

### 2.8 Statistical analysis

The experiments were performed in triplicate, and the data were expressed as mean ± SD. Student’s *t*-test was used to compare the two groups. The data of more than two groups were analyzed by one-way analysis of variance (ANOVA) using GraphPad Prism version 5 software (GraphPad Software, United States), and the threshold for significance was set at a *p*-value < 0.05.

## 3 Results

**Figure 1:**
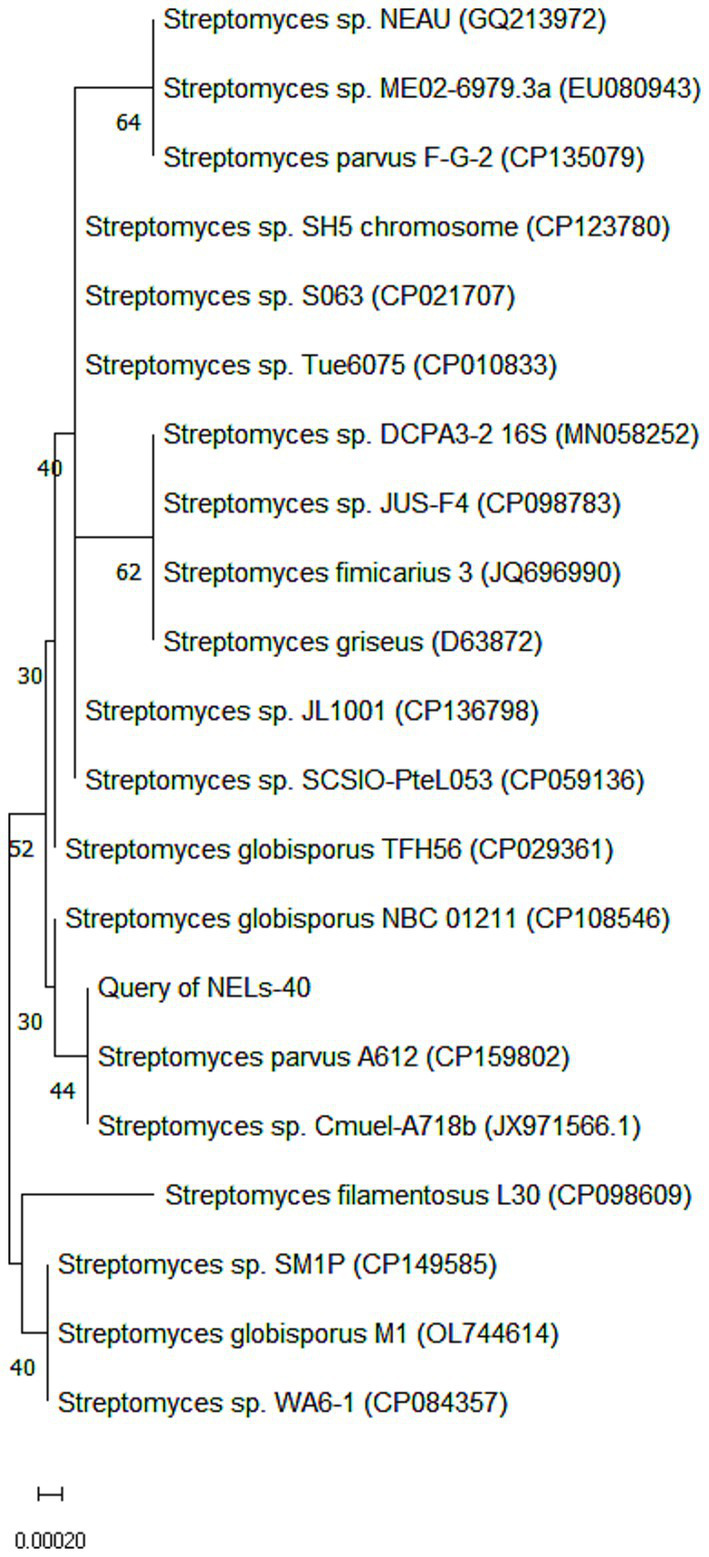
Phylogenetic tree constructed using the neighbor-joining method for strain NELs-40, derived from 16S rRNA gene sequences. The numbers on the branches represent bootstrap values from 1,000 replicates. The scale bar indicates 0.0002 nucleotide substitutions per position.

*Streptomyces* sp. strain NELs-40, isolated from mangrove soil in the coastal regions of Hainan, China, exhibited potent antimicrobial activity against diverse bacterial pathogens. After sequencing, EzBioCloud analysis revealed that the strain shares a 100% similarity with the standard strain *Streptomyces parvus*. Phylogenetic reconstruction using the neighbor-joining method positioned strain NELs-40 within a distinct subclade alongside *S. parvus*, albeit with moderate bootstrap support (44%) (Figure 1). This Whole Genome Shotgun project has been deposited at GenBank under the accession JBXATF000000000; https://www.ncbi.nlm.nih.gov/search/all/?term=JBXATF000000000. Comparative genomic analysis revealed that NELs-40 shares an Average Nucleotide Identity (ANI) of 99.6% with the *S. parvus* reference genome and 98.50% with *S. parvus* A612. Both values significantly exceed the 95–96% threshold used for prokaryotic species delineation (Richter and Rosselló-Móra, 2009). TYGS analysis (Meier-Kolthoff and Göker, 2019). Further revealed the isolate as *S. parvus*, yielding a dDDH value of 88.70% relative to the reference genome GCF_014648875.1 and 90.70% relative to the strain A612.

To further resolve the taxonomic position of strain NELs-40 at the protein level, pairwise Average Amino Acid Identity (AAI) comparisons were performed against two reference genomes including GCF_014648875.1 (*S. parvus* reference genome) and GCA_040556325.1 (*S. parvus* A612). The analysis revealed high amino acid identity between NELs-40 and both references, with AAI values of 98.31 and 98.77%, respectively (Table 1). The orthologous fractions (OF) ranged from 87.42 to 90.57%, indicating substantial proteome overlap between the strain and the reference genomes. Both AAI values substantially exceed the established 95–96% (Konstantinidis and Tiedje, 2005) species delineation threshold for prokaryotic taxonomy, providing strong protein-level evidence supporting the classification of NELs-40 as *Streptomyces parvus*.

**Table 1:** Average Amino Acid Identity (AAI) analysis of *S. parvus* strain NELs-40.

Genome A	Genes in A	Genome B	Genes in B	Orthologous	Mean AAI (%)	Std AAI	OF (%)
GCF_014648875.1	7,055	GCF_055108415.1	7,012	5,887	96.07	7.3	83.96
GCF_014648875.1	7,055	GCA_040556325.1	7,330	5,874	98.23	5.18	83.26
GCF_014648875.1	7,055	NELs-40	6,852	6,206	98.31	5.08	90.57
GCF_055108415.1	7,012	GCA_040556325.1	7,330	5,486	96.08	7.42	78.24
GCF_055108415.1	7,012	NELs-40	6,852	5,827	96.27	7.03	85.04
GCA_040556325.1	7,330	NELs-40	6,852	5,990	98.77	3.72	87.42

OF, Orthologous fraction.

The table shows pairwise average amino acid identity (AAI, %) values, standard deviations (StdAAI), and orthologous fractions (OF) among strain NELs-40 and three S. parvus genomes: GCF_014648875.1, GCA_040556325.1, and GCF_055108415.1. AAI and OF were calculated using CompareM.

### 3.1 Sequencing

#### 3.1.1 Genomic features and taxonomic classification

**Figure 2:**
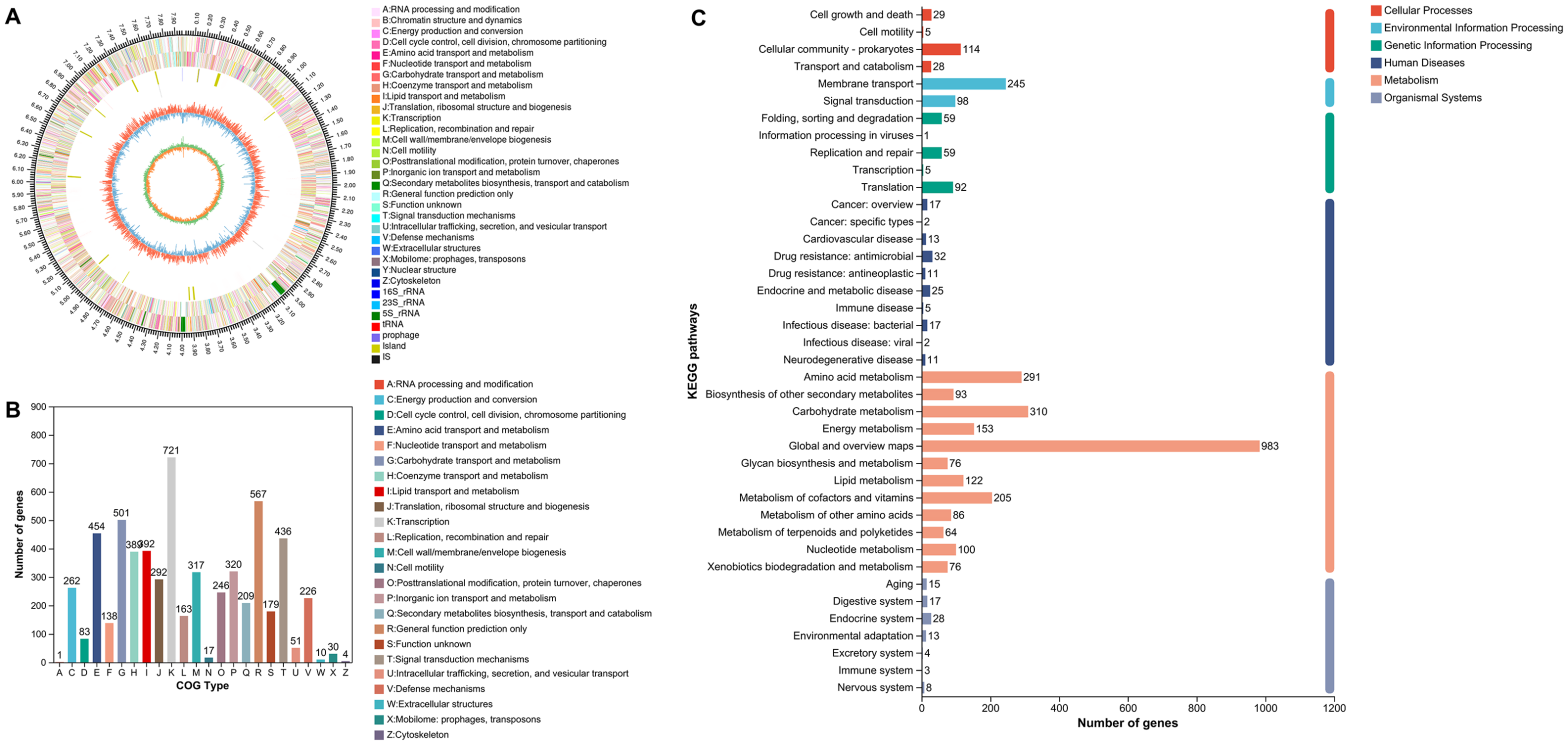
Genome sequencing and annotation of NELs-40. **(A)** Depicts a schematic representation of the NELs-40 chromosome. The outermost and fourth circles represent predicted protein-coding regions classified by COG function categories on the forward and reverse strands, with different colors indicating various COG pathway categories. Circles two and three illustrate the distribution of CDS, tRNA, and rRNA. The fifth and sixth circles display the G + C content and G + C skew. **(B)** Details of the COG classification of NELs-40 genome. **(C)** The KEGG pathway annotation.

The genome sequencing of strain NELs-40 revealed a complete genome of 7,973,786 bp with a GC content of 71.76%. The genome harbors 6,872 protein-coding genes, 2 rRNA operons, 67 tRNAs, 66 small RNAs, one prophage region, and 17 CRISPR domains (Table 2; Figure 2A).

**Table 2:** Basic features of *Streptomyces* sp. NELs-40.

Features	Value
Genome size (bp)	7,973,786
Average length of coding genes (bp)	1035.09
(G + C) %	71.76
Number of protein coding genes	6,872
Number of rRNA genes	2
Number of tRNA genes	67
CRISPR domains	17

#### 3.1.2 Annotation of gene function

All genes have been functionally annotated using several databases, including Nr (Non-Redundant Protein), GO (Gene Ontology), COG (Clusters of Orthologous Groups), and KEGG (Kyoto Encyclopedia of Genes and Genomes). Our results demonstrated that among the predicted genes, 6,657 (96.97%) were annotated in the Nr database, 5,624 (81.84%) in Pfam, 4,597 (66.89%) in Swiss-Prot, 5,349 (77.84%) in COG, 4,070 (59.23%) in KEGG, and 3,290 (47.88%) in GO databases.

KEGG pathway analysis classified 4,366 genes into six functional categories, with metabolism representing the predominant category (77.84% of coding sequences), followed by Environmental Information Processing (30.25%), Genetic Information Processing (19.71%), poorly characterized functions (15.12%), Cellular processes (12.32%), Human diseases (7.73%), and Organismal systems.

Gene Ontology (GO) database categorizes genomic information into three main categories: molecular function (M), biological process (P), and cellular component (C) in NELs-40. Three thousand two hundred and ninety genes have been annotated in the GO database, which constitutes 47.88% of all encoded genes. It mainly involved in molecular function (2,731 genes), followed by biological processes (1,573 genes) and cellular components (1,281genes), indicating that gene products are predominantly involved in molecular functions.

COG analysis classified 5,349 genes (77.84% of total genes) into functional categories, with notable distributions including: transcription (K, 13.47%, 721 genes), carbohydrate transport and metabolism (G, 9.36%, 501 genes), amino acid transport and metabolism (E, 8.48%, 454 genes), lipid transport and metabolism (I, 7.32%, 392 genes), and coenzyme transport and metabolism (H, 7.27%, 389 genes). Additionally, 10.60% of genes were assigned general functional predictions, while 3.35% remained functionally uncharacterized, warranting further investigation (Figure 2B).

Notably, 2,559 genes (96.13% of total genes) were identified as involved in metabolic regulation, highlighting the strain’s extensive metabolic capabilities (Figure 2C).

#### 3.1.3 Biosynthetic gene cluster analysis

**Figure 3:**
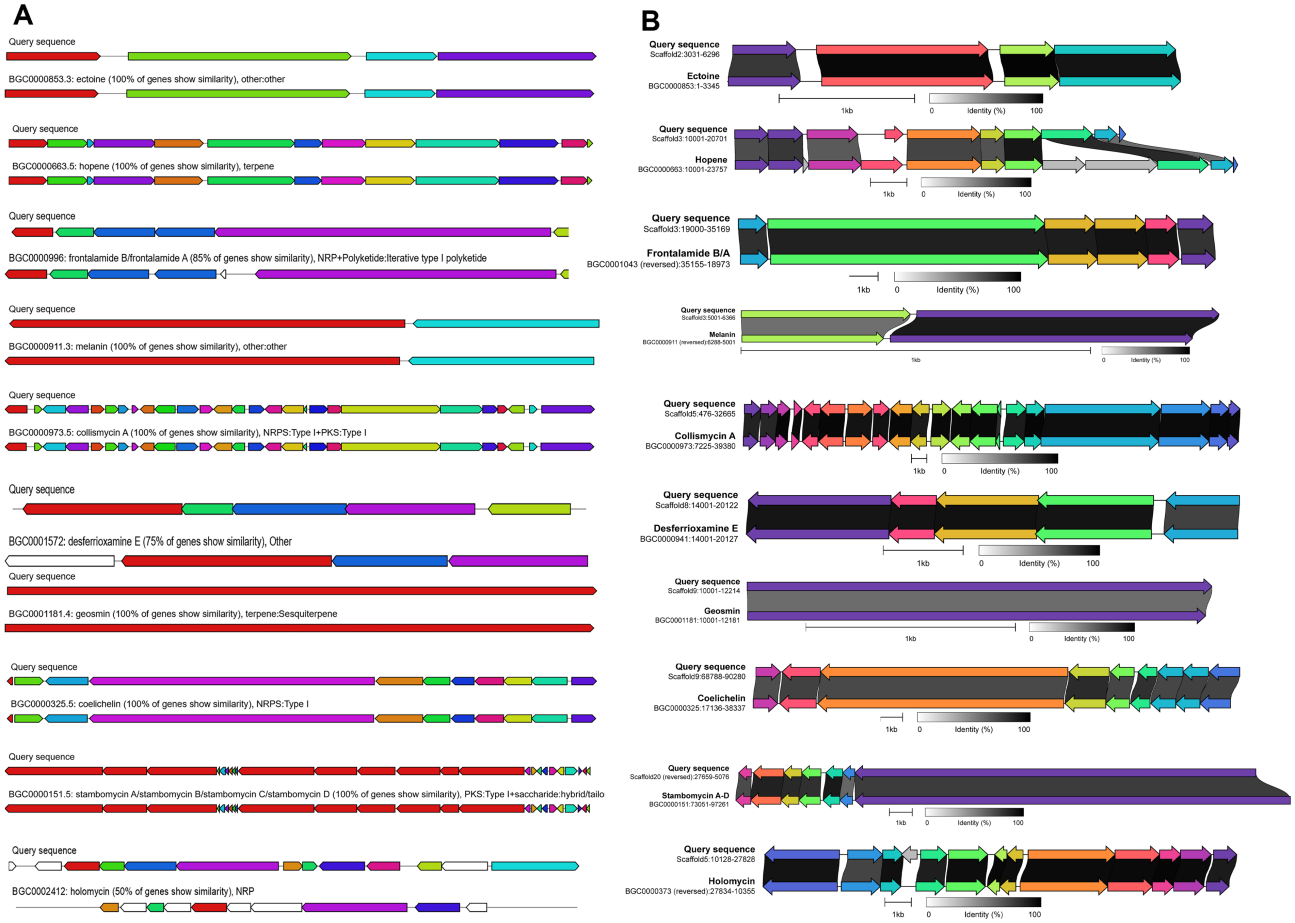
Comparative analysis of BGCs from strain NELs-40 with known clusters. **(A)** AntiSMASH comparison showing percent similarity to known clusters in the MIBiG database. **(B)** Clinker synteny plot showing collinearity and gene order conservation between NELs-40 BGCs and their homologous known clusters.

AntiSMASH analysis identified 49 biosynthetic gene clusters (BGCs) in NELs-40, demonstrating extensive secondary metabolite production capacity. These clusters are responsible for synthesizing diverse compound classes including polyketides (PKs), nonribosomal peptides (NRPs), ectoine, lantipeptides, bacteriocins, butyrolactones, indoles, terpenes, melanin, thiopeptides, siderophores, and lassopeptides. The biosynthetic repertoire comprises 17 PK clusters (types I, II, and III), 11 nonribosomal peptide synthetases (NRPSs), and three additional clusters (Table 3). Among these BGCs, 39 were annotated by aligning with the GenBank database. Six clusters exhibited over 70% similarity to known BGCs in the MIBiG database, including seven clusters with 100% similarity, for which structures and functions could be predicted (Figure 3A). Notably, many of the remaining clusters showed low similarity to known clusters, indicating substantial potential for discovering novel bioactive metabolites (Table 3). To further examine structural organization, synteny analysis was performed using Clinker v1.0.0. Comparison of selected BGCs from NELs-40 with their homologous known clusters confirm high collinearity and conserved gene order (Figure 3B), confirming structural conservation and functional authenticity.

**Table 3:** AntiSMASH predicted BGCs from *Streptomyces* sp. NELs-40.

BGC	Position (From–To)	Product/type	Predicted compound	Similarity (%)	Category
cluster1	162,979–183,987	Terpene	–	0	Unannotated
cluster2	305,929–316,304	Ectoine	Showdomycin	35	Low
cluster3	564,141–586,955	lanthipeptide-class-III	AmfS	100	High
cluster4	677,845–732,546	T1 PKS	EnduracidinA/B	8	Low
cluster5	68,569–78,968	Ectoine	Ectoine	100	High
cluster6	119,466–197,546	PKS-like	fluostatin M/N/O/P/Q	16	Low
cluster7	348,108–374,830	Terpene	Hopene	69	Medium
cluster8	447,769–498,310	NRPS	SGR PTMs	100	High
cluster9	614,399–662,364	T1 PKS	Valinomycin; montanastatin	13	Low
cluster10	696,372–706,855	Melanin	Melanin	100	High
cluster11	717,114–745,544	Thiopeptide	Lactazole	33	Low
cluster12	749,805–853,624	T3 PKS	Tetronasin	11	Low
cluster13	916,930–969,092	T1 PKS	Kanamycin	2	Low
cluster14	447,213–473,696	Lanthipeptide-Class-II	Chalcomycin A	9	Low
cluster15	701,015–789,781	NRPS	Skyllamycin A/B	44	Low
cluster16	56,094–70,844	Siderophore	Ficellomycin	3	Low
cluster17	285,194–334,770	T1 PKS	Collismycin A	74	High
cluster18	374,120–385,449	RiPP-like	–	0	Unannotated
cluster19	400,273–443,540	NRPS	Holomycin	92	High
cluster20	630,870–652,181	Terpene	Formicamycin A/B/C	16	Low
cluster21	64,180–96,671	LAP	–	0	Unannotated
cluster22	176,455–216,812	NRPS-like	–	0	Unannotated
cluster23	351,054–405,496	NRPS	Phosphonoglycans	3	Low
cluster24	414,904–437,518	Lassopeptide	–	0	Low
cluster25	74,875–95,952	Terpene	Steffimycin D	19	Low
cluster26	263,173–274,952	Siderophore	Desferrioxamin B	100	High
cluster27	336,473–367,650	Lanthipeptide-class-III	–	0	Unannotated
cluster28	714–43,484	NRPS-like	Nocathiacin	4	Low
cluster29	61,634–72,579	Butyrolactone	Coelimycin P1	16	Low
cluster30	103,575–125,789	Terpene	Geosmin	100	High
cluster31	159,416–204,355	NRPS	Griseobactin	94	High
cluster32	209,292–260,278	NRPS	Coelichelin	81	High
cluster33	190,631–219,609	Other	Daptomycin	18	Low
cluster34	39,651–80,770	T3PKS	Herboxidiene	6	Low
cluster35	82,368–105,251	Banthipeptide-class-IV	Labyrinthopeptin A1/A2/A3	40	Low
cluster36	38,031–60,845	Lassopeptide	Keywimysin	100	High
cluster37	1–27,689	NRPS-like	Malacidin A/B	5	Low
cluster38	1–27,659	T1 PKS	Stambomycin A/B/C/D	60	Medium
cluster39	1–19,372	T1 PKS	Lydicamycin	24	Low
cluster40	1–15,659	T1 PKS	Chalcomycin A	12	Low
cluster41	1–10,851	T1 PKS	Stambomycin A/B/C/D	36	Low
cluster42	1–8,468	T1 PKS	Piericidin A1	41	Low
cluster43	1–8,099	T1 PKS	Azalomycin F3a	34	Low
cluster44	1–7,751	T1 PKS	–	0	Unannotated
cluster45	1–5,651	T1 PKS	Nanchangmycin	30	Low
cluster46	1–4,183	T1 PKS	ECO-02301	32	Low
cluster47	1–4,061	T1 PKS	–	0	Unannotated
cluster48	1–2,370	NRPS	–	0	Unannotated
cluster49	1–1,266	NRPS	–	0	Unannotated

Genome analysis further revealed that 30 gene clusters were specifically associated with antimicrobial compound biosynthesis, including showdomycin, AmfS, enduracidin, fluostatins M-Q, valinomycin, montanastatin, lactazole, tetronasin, kanamycin, chalcomycin A, RP-1776, ficellomycin, collismycin A, holomycin, formicamycins A-M, phosphonoglycans, steffimycin D, nocathiacin, coelimycin P1, daptomycin, herboxidiene, labyrinthopeptin variants (A1, A2, A3), malacidin variants (A, B), stambomycin variants (A, B, C, D), lydicamycin, piericidin A1, azalomycin F3a, nanchangmycin, and ECO-02301. Three BGCs were dedicated to siderophore production (ficellomycin, coelichelin, and desferrioxamine B) for iron chelation and environmental iron homeostasis. The presence of numerous uncharacterized BGCs suggests significant potential for discovering novel bioactive metabolites (Gu et al., 2020).

### 3.2 Antibacterial activity of NELs-40

#### 3.2.1 Culture medium optimization for antimicrobial production

*Streptomyces* sp. strain NELs-40 demonstrated variable antimicrobial production across different liquid culture media tested over 7 days. The strain exhibited optimal activity in TSB (29.84 ± 2.13 mm) and YEME broth (28.16 ± 3.42 mm) against MRSA ATCC 43300 (Table 4). Moderate production was observed in SCA broth (26.73 ± 2.67 mm) and potato dextrose broth (25.94 ± 2.89 mm), while Bennett’s broth (24.38 ± 1.94 mm) and nutrient broth (23.85 ± 4.16 mm) showed comparable performance. YM broth (21.67 ± 2.45 mm) and glucose-yeast extract broth (18.29 ± 2.18 mm) yielded the lowest activities. Statistical analysis revealed significant differences between media types (*p <* 0.05), with substantial variation indicating that NELs-40’s secondary metabolite production is highly responsive to nutritional composition. These findings established TSB as the optimal basal medium for subsequent optimization studies.

**Table 4:** Antibacterial activity of *Streptomyces* sp. NELs-40 cultured in different culture media against MRSA ATCC 43300, measured by inhibition zone diameter.

Culture medium	Antibacterial activity (mm)
TSB (tryptic soy broth)	29.84 ± 2.13^a^
YEME broth	28.16 ± 3.42^ab^
SCA broth (starch casein)	26.73 ± 2.67^bc^
Potato dextrose broth	25.94 ± 2.89^bc^
Bennett’s broth	24.38 ± 1.94^cd^
Nutrient broth	23.85 ± 4.16^cd^
YM broth (yeast-malt)	21.67 ± 2.45^de^
Glucose-yeast extract broth	18.29 ± 2.18^ei^

Values represent mean ± standard deviation (mm) of inhibition zones. Different letters indicate statistically significant differences between culture media (*P <* 0.05).

#### 3.2.2 Statistical optimization of antimicrobial metabolites

Plackett-Burman design was employed to optimize bioactive compound production by NELs-40. The design matrix of 12 runs with five variables yielded inhibition zones ranging from 16.28 to 37.82 mm against MRSA ATCC 43300 (Supplementary Table S3). The regression model was expressed as:

Y=+25.53+0.94A–3.92B+2.32C+2.59D+0.73E

Where *Y* represents antibacterial activity, and *A*, *B*, *C*, *D*, and *E* are coded values for temperature, pH, inoculation time, agitation speed, and inoculum size, respectively.

ANOVA analysis demonstrated marginal model significance (*F* = 4.66, *p* = 0.0522) with pH (B) identified as the only significant factor (*p* < 0.05). Factors C and D showed marginal significance (*p* = 0.08 and *p* = 0.06, respectively). The model showed moderate reliability with *R*^2^ = 0.7951, adjusted *R*^2^ = 0.6243, predicted *R*^2^ = 0.3158, and coefficient of variation = 15.15% (Tables 5, 6). The validation study confirmed the importance of pH as the primary factor affecting antimicrobial production, while demonstrating realistic biological variation typical of fermentation processes.

**Table 5:** Analysis of the data generated by the Plackett–Burman design.

Factor	Effect	Coefficient	Standard error	*P* value	Significance
Intercept		25.45	0.89	0.0000	
A	1.87	0.94	0.89	0.3214	
B	−7.84	−3.92	0.89	0.0019	**
C	4.63	2.32	0.89	0.0286	*
D	5.18	2.59	0.89	0.0178	*
E	1.45	0.73	0.89	0.4387	

**Table 6:** Results of the ANOVA for the regression equation.

Source	Sum of square	df	Mean square	*F* value	*p*-value prob.>F	Significance
Model	348.23	5	69.65	4.66	0.0522	Significant
Residual	89.74	6	14.96			
A	10.60	1	10.60	0.71	0.4299	
B	184.40	1	184.40	12.33	0.0124	
C	64.59	1	64.59	4.32	0.0831	
D	80.50	1	80.50	5.52	0.0599	
E	6.39	1	6.39	0.43	0.5376	
Cor Total	437.97	11				

SD = 3.87.

Mean = 25.53.

Coefficient of determination *R*^2^ = 0.7851.

Adjusted coefficient of determination *R*^2^ = 0.6243.

Predicted coefficient of determination *R*^2^ = 0.3158.

Coefficient of variation = 15.15%.

Adequate precision = 6.842.

#### 3.2.3 Antibacterial efficacy of NELs-40 extract

**Figure 4:**
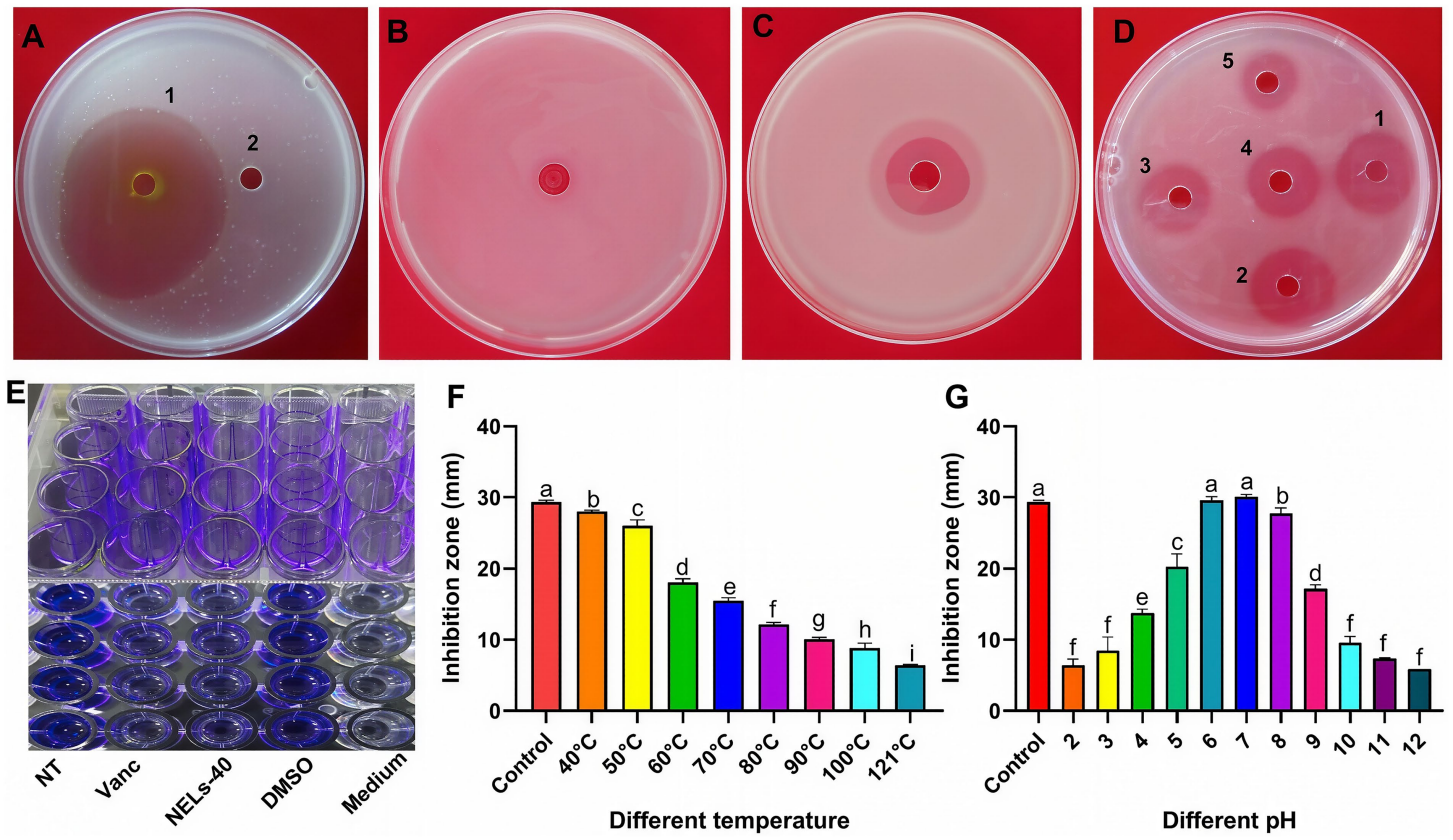
Antibacterial activity of *Streptomyces parvus* strain NELs-40 against *Staphylococcus aureus* ATCC 43300. **(A)** Agar well diffusion assay showing inhibition zones produced by the NELs-40 extract against the tested bacterial pathogen (1: NELs-40 extract, 2: extract from sterile medium without bacterial inoculation). **(B)** DMSO solvent control showing no antibacterial activity. **(C)** Vancomycin positive control. **(D)** Residual antibacterial activity of the extract following exposure to various enzymes (1: control, 2: trypsin, 3: proteinase K, 4: lipase, 5: pepsin). **(E)** Biofilm inhibition assay in microtiter plates stained with crystal violet, demonstrating a reduction in biofilm biomass in the presence of the NELs-40 extract compared to untreated controls. **(F)** Heat stability of the extract, measured by residual activity after exposure to different temperatures. **(G)** pH stability of the extract, determined by residual activity after incubation at varying pH levels.

The ethyl acetate extract of NELs-40 exhibited potent broad-spectrum antibacterial activity MRSA ATCC 43300, producing inhibition zones of 37 mm and demonstrating significant therapeutic potential against the test organism (Figure 4A). The antimicrobial efficacy was further validated through minimum inhibitory concentration (MIC) determination, revealing effective bacterial growth inhibition at concentrations as low as 125 μg/mL. Neither the negative control nor the DMSO solvent control exhibited any anti-biofilm activity (Figures 4A2,B), while the positive control vancomycin produced a 24 mm inhibition zone in the agar well diffusion assay (Figure 4C).

Anti-biofilm activity assessment revealed that the NELs-40 extract at 2 mg/mL significantly impaired MRSA ATCC 43300 biofilm formation on polystyrene surfaces. Based on four replicates, the extract reduced biofilm biomass by 32.25% compared to untreated controls (Figure 4E). The positive control vancomycin achieved a reduction of 88.7%, while the DMSO solvent control exhibited no anti-biofilm activity.

Stability characterization of the NELs-40 extract revealed significant temperature-dependent activity retention (*F* = 1159.82, *p* < 0.001). The extract maintained 95.5% activity at 40 °C but showed progressive thermal degradation at higher temperatures, with critical activity loss beginning at 60 °C (61.6% retention). At 70 °C, only 52.7% activity remained, while exposure to 100 °C and 121 °C resulted in 69.8 and 78.1% activity losses, respectively, following 30-min incubation periods (Figure 4F). pH stability analysis identified optimal activity within pH 6.0–7.0 range, with these conditions being statistically equivalent (*p* > 0.05). Diminished antimicrobial efficacy was observed under both acidic (pH < 6.0) and alkaline (pH > 8.0) conditions, with extreme pH values (≤5.0 or ≥9.0) causing 67–80% activity reduction (Figure 4G). Duncan’s Multiple Range Test confirmed six distinct statistical groups, demonstrating high pH sensitivity beyond the neutral range. Notably, the extract demonstrated remarkable resistance to proteolytic degradation by trypsin and lipase, suggesting the bioactive compounds possess non-proteinaceous characteristics and structural stability against enzymatic hydrolysis (Figure 4D).

### 3.3 GC–MS identification of bioactive compounds

GC–MS analysis of NELs-40 extracts revealed 23 distinct chemical compounds identified through NIST library matching, with their chemical structures and functional classifications documented (Table 7). The extract composition was dominated by 9-octadecenoic acid, methyl ester, (E)-, which constituted the largest single component at 100% relative peak area. Other significant contributors included pyrrolo[1,2-a]pyrazine-1,4-dione derivatives (45.83 and 26.34%), caryophyllenyl alcohol (41.17%), and tyrosol acetate (35.89%). Ammonium acetate represented a substantial portion at 80.46%, while α-calacorene contributed 26.46% to the total composition. The identified compounds encompassed diverse chemical classes including esters (representing the majority), alcohols, amides, sesquiterpenes, alkanes, and heterocyclic compounds. Fatty acid esters, particularly long-chain unsaturated esters, were prominently featured, suggesting potential antimicrobial and bioactive properties characteristic of microbial secondary metabolites.

**Table 7:** Compounds identified from NELs-40 extract using GC–MS.

No.	RT	Compound name	Type	Area (%)	Compound structure
1	2.977	1,4-Dioxane-2,5-dione, 3,6-dimethyl-	Ester	10.08	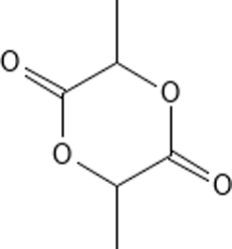
2	4.231	Ammonium acetate	Organic acid	80.46	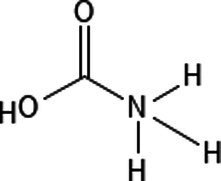
3	5.005	Acetamide	Amide	4.86	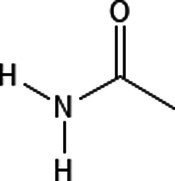
4	5.302	Pyridine, 2,3,4,5-tetrahydro-	Amine	0.21	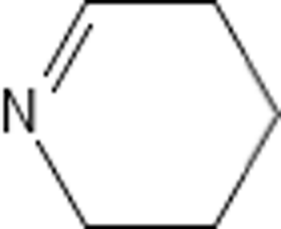
5	10.318	2-Pyrrolidinone	Amide	1.63	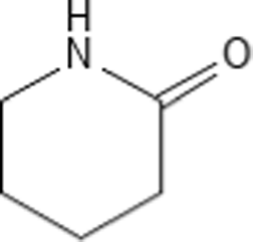
6	11.129	Phenylethyl Alcohol	Alcohol	2.27	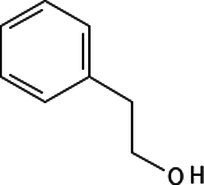
7	11.898	6-Methyl-2-pyrazinylmethanol	Alcohol	0.14	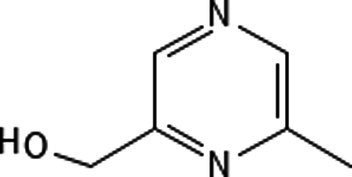
8	14.781	2-Piperidinone	Amide	2.36	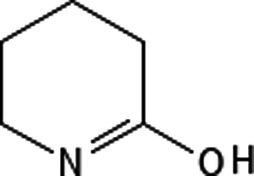
9	15.582	5-Thiazoleethanol, 4-methyl-	Alcohol	0.79	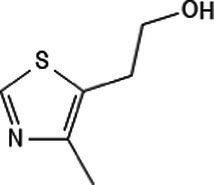
10	15.983	m-Aminophenylacetylene	Amine	0.77	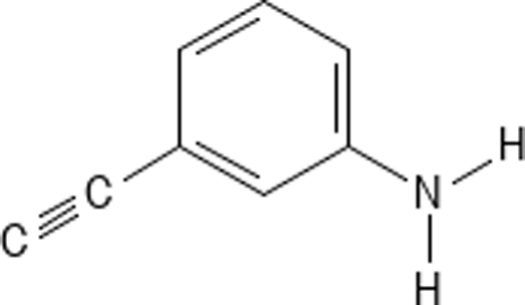
11	17.713	Benzeneacetic acid, 2-hydroxy-, methyl ester	Ester	0.76	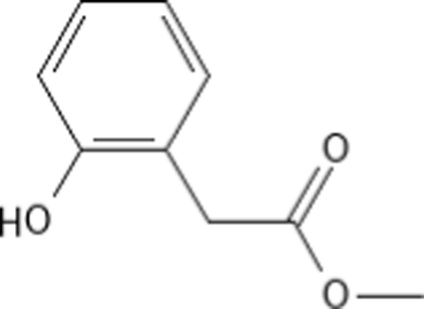
12	18.761	Benzeneacetamide	Amide	3.7	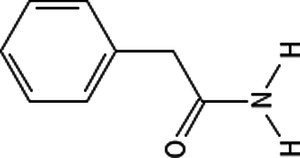
13	22.912	Caryophyllenyl alcohol	Alcohol	0.96	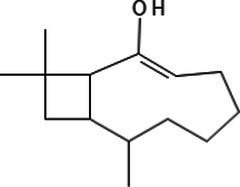
14	23.056	Di-epi-1,10-cubenol	Alcohol	20.24	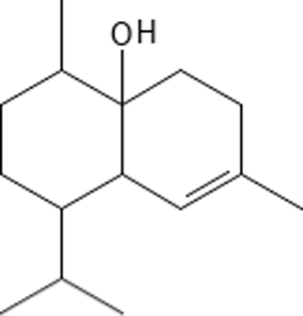
15	25.544	Hexadecanoic acid, methyl ester	Fatty acid ester	4.18	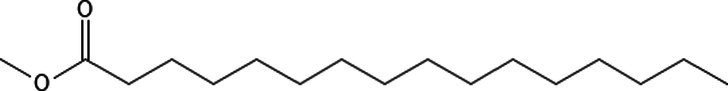
16	26.172	Docosane	Alkane	8.52	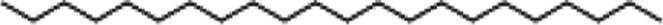
17	27.742	α-Calacorene	Sesquiterpene	26.46	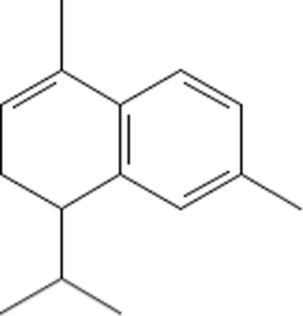
18	29.122	Tyrosol, acetate	Ester	35.89	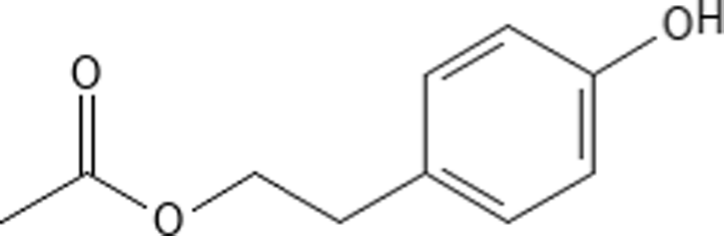
19	30.37	Phthalic acid, di(2-propylpentyl) Ester	Ester	23.69	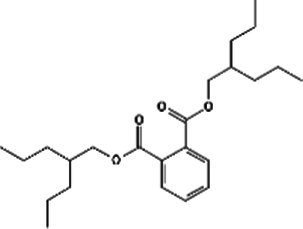
20	30.878	Caryophyllenyl alcohol	Alcohol	41.17	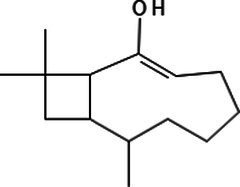
21	31.303	9-Octadecenoic acid, methyl ester, (E)-	Ester	100	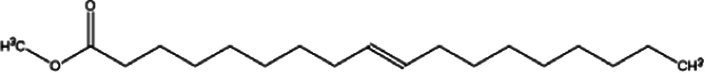
22	37.948	Pyrrolo[1,2-a]pyrazine-1,4-dione, hexahydro-3-(phenylmethyl)-	Heterocyclic compound	45.83	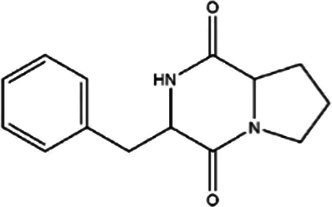
23	38.120	Cyclo-(l-leucyl-l-phenylalanyl)	Diketopiperazine	1.1	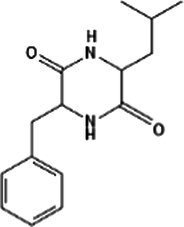
24	38.567	Pyrrolo[1,2-a]pyrazine-1,4-dione, hexahydro-3-(phenylmethyl)-	Heterocyclic compound	26.34	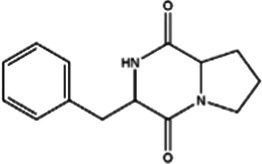

## 4 Discussion

This study presents a comprehensive characterization of *Streptomyces* sp. strain NELs-40, a marine-derived actinomycete isolated from mangrove rhizosphere soil, demonstrating its remarkable potential as a source of novel antimicrobial compounds. Through integrated genomic, chemical, and bioactivity analyses, we reveal that NELs-40 possesses exceptional biosynthetic capabilities, produces potent antimicrobial metabolites effective against drug-resistant pathogens, and harbors numerous uncharacterized biosynthetic gene clusters with significant capacity for bioactive compound discovery.

The comprehensive genomic characterization of Streptomyces sp. strain NELs-40 provides compelling evidence for its classification as *S. parvus*, supported by an ANI value of 99.6% that exceeds the established species delineation threshold (Jain et al., 2018). This classification was further supported by TYGS analysis, which yielded dDDH values of 88.70% relative to the reference genome GCF_014648875.1 and 90.70% relative to strain A612, both well above the 70% species threshold. At the protein level, pairwise Average AAI analysis yielded values of 98.31 and 98.77% against two reference genomes, also both exceeding the 95 to 96% AAI threshold for species delineation. Collectively, these multiple lines of phylogenomic evidence including ANI, dDDH, and AAI classify NELs-40 as *S. parvus*.

The complete genome (7,973,786 bp, 71.76% GC content) contains 6,872 protein-coding genes with high proportions of metabolism-related (77.84%) and metabolic regulation genes (96.13%), demonstrating sophisticated regulatory networks characteristic of Streptomyces complexity (Bentley et al., 2002; Lee et al., 2020; Khushboo et al., 2023). AntiSMASH analysis revealed 49 biosynthetic gene clusters (BGCs), positioning NELs-40 among the most prolific secondary metabolite producers (Blin et al., 2023). Notably, 30 of these clusters are specifically associated with antimicrobial biosynthesis, suggesting their natural ecological roles in microbial competition (van der Meij et al., 2017; Jose et al., 2021; Khushboo et al., 2023). Synteny analysis using Clinker revealed high collinearity and conserved gene cluster organization between NELs-40 and closely related *S. parvus* reference genomes, further supporting their close evolutionary relationship (Gilchrist and Chooi, 2021). The diversity of predicted metabolites encompasses polyketides, nonribosomal peptides, and both characterized antibiotics (kanamycin, daptomycin) and novel compounds like malacidin variants. Multiple siderophore-producing BGCs (ficellomycin, coelichelin, desferrioxamine B) demonstrate marine adaptation through iron acquisition mechanisms that likely contribute to antimicrobial activity via nutrient sequestration. The low similarity of many BGCs to known clusters indicates substantial potential for novel bioactive discovery, providing a valuable foundation for next-generation therapeutic development against multidrug-resistant bacterial infections. The comprehensive genomic characterization enables targeted genetic engineering approaches to enhance production yields, activate silent clusters, and optimize bioactive compound biosynthesis through precise manipulation of identified regulatory genes and biosynthetic pathways.

Building upon this genomic foundation, the statistical optimization using Plackett-Burman design identified pH as the primary significant factor (*p* < 0.05) affecting antimicrobial production by NELs-40. The negative coefficient for pH (−3.92) suggests that lower pH values favor antimicrobial production, consistent with acidic conditions preferred by many *Streptomyces* species for antibiotic synthesis (Manikandan et al., 2019). Inoculation time and agitation speed showed marginal significance, indicating their potential secondary importance in the production process. The model’s moderate reliability (*R*^2^ = 0.7951, adjusted *R*^2^ = 0.6243) indicates that approximately 80% of the variation in antimicrobial activity is explained by the tested factors (Selvaraj et al., 2022). The moderate predicted *R*^2^ (0.3158) and coefficient of variation (15.15%) reflect inherent biological variation typical of fermentation processes, while the marginal model significance (*F* = 4.66, *p* = 0.0522) emphasizes the complex nature of microbial secondary metabolite production (Wang et al., 2011).

In the agar well diffusion assay at an equivalent concentration of 2 mg per well, the NELs-40 extract produced a larger inhibition zone (37 mm) against *S. aureus* ATCC 43300 compared to vancomycin (24 mm). This may be partially explained by the presence of volatile compounds detected by GC–MS, which diffuse more rapidly through agar than the larger vancomycin molecule (~1,450 Da). However, the MIC of the crude extract (125 μg/mL) was substantially higher than the reported MIC of vancomycin against this reference strain (~1–1.5 μg/mL) (Zhu et al., 2012; Entenza et al., 2014). This apparent discrepancy reflects fundamental differences between the two assays. The agar diffusion assay measures both potency and diffusion rate, while the MIC assay provides a direct measure of intrinsic potency in a diffusion-free liquid medium. The higher MIC of the crude extract is expected, as the active compound(s) constitute only a fraction of the total dry weight. Following purification, the MIC of the isolated active compound(s) may decrease substantially. Collectively, these findings confirm the presence of potent bioactive compounds in the NELs-40 extract with favorable diffusion properties, while highlighting the importance of further purification to fully realize their therapeutic potential.

Chemical analysis through GC–MS revealing 23 distinct compounds provides valuable mechanistic insights into NELs-40’s antimicrobial activity. The dominance of 9-octadecenoic acid methyl ester, pyrrolo[1,2-a]pyrazine-1,4-dione derivatives, and sesquiterpenes suggests a synergistic mechanism of action. Long-chain fatty acid esters possess membrane-disrupting properties contributing to antimicrobial efficacy (Ye et al., 2024; Zhang et al., 2025), while pyrrolo[1,2-a]pyrazine derivatives are particularly noteworthy for their established antibacterial (Singh et al., 2019), antifungal (Awan et al., 2023; Mahmoud et al., 2023), and antioxidant activities (Ser et al., 2015). This diverse chemical composition indicates multiple mechanisms of action that reduce rapid resistance development compared to single-target antibiotics (Silver, 2007), explaining the broad-spectrum activity and potency exceeding 37 mm inhibition zones observed against drug-resistant *Staphylococcus* strains. However, it should be noted that GC–MS analysis is limited to the detection of volatile and semi-volatile compounds, and compound identification based on spectral library matching is tentative. Non-volatile, thermally labile metabolites, or high molecular weight metabolites would not be detected under standard GC–MS conditions. Therefore, the results may not fully represent all bioactive metabolites, and further confirmation using complementary techniques such as LC–MS or NMR is required for definitive identification.

This comprehensive study demonstrates that *Streptomyces* sp. strain NELs-40 represents an exceptional resource for combating antibiotic resistance, with extensive biosynthetic gene cluster diversity, potent activity against drug-resistant pathogens, and chemical complexity supporting its development as a source of novel antimicrobial agents. The detailed characterization of bioactive compounds and genomic foundation provide a platform for future drug discovery efforts and rational strain improvement strategies.

## Data Availability

The data presented in the study are available in the NCBI GenBank repository, accession number JBXATF000000000 (https://www.ncbi.nlm.nih.gov/nuccore/JBXATF000000000).
