# Supplementary material for: Characterization and antibacterial efficacy of Streptomyces sp. NELs-40 against Staphylococcus aureus
Source: Front Microbiol. 2026 May 29;17:1840366. doi: 10.3389/fmicb.2026.1840366 (PMC13260292; doi:10.3389/fmicb.2026.1840366)
Supplement: Supplementary file 1 [file Table_1.docx]

**Table S1.** Culture medium used in this study

| Medium | Composition/liter |
| --- | --- |
| TSB | Purchased from Hopebio, Qingdao, China |
| LB | Tryptone 10 g; yeast extract 5 g; NaCl 10 g (agar 15–20 g for solid medium) |
| YEME broth | Yeast extract 3 g; malt extract 3 g; glucose 10 g; sucrose 340 g |
| SCA broth | Soluble starch 10 g; casein 0.3 g; K₂HPO₄ 2 g; MgSO₄·7H₂O 0.05 g; NaCl 2 g; FeSO₄·7H₂O 0.01 |
| Potato dextrose broth | Potato infusion (from 200 g potatoes); dextrose 20 g |
| Bennett’s broth | Beef extract 1 g; yeast extract 1 g; peptone 2 g; glucose 10 g |
| Nutrient broth | Beef extract 3 g; peptone 5 g; NaCl 5 g |
| YM broth (Yeast-Malt) | Yeast extract 3 g; malt extract 3 g; peptone 5 g; glucose 10 g |
| Glucose-yeast extract broth | Glucose 10 g; yeast extract 5 g |
| Medium | Composition/liter |
