# Supplementary material for: Characterization and antibacterial efficacy of Streptomyces sp. NELs-40 against Staphylococcus aureus
Source: Front Microbiol. 2026 May 29;17:1840366. doi: 10.3389/fmicb.2026.1840366 (PMC13260292; doi:10.3389/fmicb.2026.1840366)
Supplement: Supplementary file 2 [file Table_2.docx]

**Table S2.** variables selected for Plackett–Burman design

| Factors | Codes | Levels | |
| --- | --- | --- | --- |
|  |  | Low level (-1) | High level (+1) |
| Temperature | A | 25 °C | 42 °C |
| pH | B | 5.0 | 8.0 |
| Inoculation time | C | 120 rpm | 200 rpm |
| Agitation speed | D | 5 days | 17 days |
| Inoculation size | E | 1% | 3% |

*− 1, minimum range of variables; + 1, maximum range of variable*
