# Supplementary material for: Characterization and antibacterial efficacy of Streptomyces sp. NELs-40 against Staphylococcus aureus
Source: Front Microbiol. 2026 May 29;17:1840366. doi: 10.3389/fmicb.2026.1840366 (PMC13260292; doi:10.3389/fmicb.2026.1840366)
Supplement: Supplementary file 3 [file Table_3.docx]

**Table S3.** The applied Plackett–Burman experimental design for five culture variables

| Trials | Variables | | | | | Antimicrobial activity (mm) | |
| --- | --- | --- | --- | --- | --- | --- | --- |
|  | A | B | C | D | E | Actual  values | Predicted values |
| 1 | +1 | +1 | +1 | -1 | -1 | 21.34 | 20.85 |
| 2 | +1 | +1 | -1 | -1 | -1 | 16.28 | 15.73 |
| 3 | -1 | -1 | -1 | -1 | -1 | 23.87 | 24.41 |
| 4 | +1 | -1 | -1 | -1 | +1 | 26.94 | 27.65 |
| 5 | +1 | -1 | +1 | +1 | -1 | 34.16 | 35.23 |
| 6 | +1 | -1 | +1 | +1 | +1 | 37.82 | 37.47 |
| 7 | -1 | +1 | -1 | +1 | +1 | 20.47 | 21.93 |
| 8 | -1 | -1 | +1 | -1 | +1 | 29.62 | 30.17 |
| 9 | -1 | +1 | +1 | -1 | +1 | 19.15 | 20.41 |
| 10 | -1 | +1 | +1 | +1 | -1 | 24.28 | 24.99 |
| 11 | +1 | +1 | -1 | +1 | +1 | 23.54 | 22.17 |
| 12 | -1 | -1 | -1 | +1 | -1 | 27.91 | 28.75 |
